# Supplementary material for: Genomic organization, gene expression and activity profile of Marinobacter hydrocarbonoclasticus denitrification enzymes
Source: PeerJ. 2018 Sep 21;6:e5603. doi: 10.7717/peerj.5603 (PMC6152468; doi:10.7717/peerj.5603)
Supplement: Supplemental Information 1 [file peerj-06-5603-s001.pdf]

Supplementary Information

Genomic organization, gene expression and activity profile of *Marinobacter hydrocarbonoclasticus* denitrification enzymes

Cíntia Carreira[1,2], Olga Mestre[1], Rute F. Nunes[1], Isabel Moura[2], Sofia R. Pauleta*[1]

[1] Microbial Stress Lab, UCIBIO, REQUIMTE, Departamento de Química, Faculdade de Ciências e Tecnologia, Universidade Nova de Lisboa, Campus da Caparica, 2829-516 Caparica, Portugal.
[2] Biological Chemistry Lab, LAQV, REQUIMTE, Departamento de Química, Faculdade de Ciências e Tecnologia, Universidade Nova de Lisboa, Campus da Caparica, 2829-516 Caparica, Portugal.

Corresponding Author
Sofia R. Pauleta
Microbial Stress Lab, UCIBIO, REQUIMTE, Departamento de Química, Faculdade de Ciências e Tecnologia, Universidade Nova de Lisboa, Campus da Caparica, 2829-516 Caparica, Portugal.
Email: srp@fct.unl.pt
Tel. +351 212 948 385, ext. 10967
Fax: + 351 212 948 550

Index

S1 - List of denitrification genes identified in different bacteria S2
S2 – Bioinformatic analysis of denitrification gene cluster of *M. hydrocarbonoclasticus* S2
S3 – Score of the alignments of FNR and IHF motifs S4
S4 – Composition of the Starkey oligoelement solution S4
S5 – Oxygen profile and schematic representation of the bioreactor S4
S6 – NO and N_2O reduction by the whole-cells - Data Analysis S5
S7 – Genome organization of *M. hydrocarbonoclasticus* denitrification genes S5

S1 – List of denitrification genes identified in different bacteria

In Table S1 are listed the genes that have been identified to encode catalytic enzymes/subunits that catalyze the different steps of the denitrification pathway.

Table S1 – Genes that encode the catalytic subunits of the enzymes involved in the denitrification pathway.

Step	Enzyme	Gene encoding the catalytic subunit	Ref.
$NO_3^- \rightarrow NO_2^-$	Membrane nitrate reductase (NaR)	*narG*	(Ghiglione et al. 1999)
	Periplasmic nitrate reductase (NaP)	*napA*	(Gates et al. 2003)
$NO_2^- \rightarrow NO$	Nitrite reductase cytochrome cd_1 (cd_1NiR)	*nirS*	(Jungst et al. 1991)
	Copper nitrite reductase (CuNiR)	*nirK*	(Cantera & Stein 2007)
	Three domain T1Cu_CuNiR*	*Cu_nirK*	(Nojiri et al. 2007)
	Three domain cyt*c*_CuNiR**	*Cyt_nirK*	(Ellis et al. 2007)
$NO \rightarrow N_2O$	Cytochrome *c* nitric oxide reductase (*c*-NOR)	*cnorB*	(Arai et al. 1995)
	Quinol nitric oxide reductase (*q*-NOR)	*qnorB (norZ)*	(Cramm et al. 1999; Hendriks et al. 2000)
	CuA nitric oxide reductase (Cu_A-NOR)	*Not identified*	(Al-Attar & de Vries 2015)
$N_2O \rightarrow N_2$	Nitrous oxide reductase (N_2OR)	*nosZ*	(Viebrock & Zumft 1988)
	Cytochrome *c* nitrous oxide reductase (*c*N_2OR)	*cnosZ*	(Simon et al. 2004)

Notes: * This is a hexameric nitrite reductase isolated from *Hyphomicrobium denitrificans* that contains an additional N-terminal cupredoxin domain. ** The gene coding for this enzyme has been identified at the genome level and is proposed to present an additional C-terminal class I *c*-type cytochrome domain.

S2 - Bioinformatic analysis of denitrification gene cluster of *M. hydrocarbonoclasticus*

The identification of putative gene functions was performed by BLAST search using the web platforms of blastp and blastn suites (NCBI) (Altschul et al. 1997) (statistic data are provided in Table S1), gene and protein alignments using ClustalOmega (McWilliam et al. 2013), and comparison of gene organization with other denitrifying microorganisms.

Table S2 - Summary of the statistics for the homology of the gene clusters proposed to be involved in the denitrification of *Marinobacter hydrocarbonoclasticus*. The score is given for the higher score between each encoded protein in a search that only included the genomes of *Pseudomonas stutzeri* str. ZoBell 632 (taxid: 32042; Ps), *Pseudomonas aeruginosa* PAO1 (taxid:208964; Pa) and *Paracoccus denitrificans* PD1222 (taxid: 318586; Pd).

Gene ID	Gene designation	Identity (%); organism	E-value	Coverage (%)
MARHY3014	*c-norB*	27, Pd	3e-25	61
	*q-nor*** (WP_055357926.1)	37, Gs	6e-163	95
MARHY3015	*moaA*	65, Ps	2e-152	98
MARHY3016	*nosL*	50, Ps	1e-57	92
MARHY3017	*nosY*	67, Ps	4e-105	99
MARHY3018	*nosF*	57, Ps	4e-107	97
MARHY3019	*nosD*	58, Pa	9e-159	85
MARHY3020	*nosZ*	76, Pa	0	97
MARHY3021	*nosR*	62, Ps	0	99
MARHY3022	Hypothetical protein (WP_003282777.1)	50, Ps	2e-44	99
MARHY3023	*dnr*	36, Ps	5e-39	86
MARHY3024	*Peptidylpropyl isomerase*	43, Ps	4e-57	93
MARHY3025	*narV, nar gamma*	57, Pd	1e-83	98
MARHY3026	*narJ,*	48, Pa	1e-57	78
MARHY3027	*narH (beta)*	81, Pa	0	98
MARHY3028	*narG (alpha)*	75, Ps	0	98
MARHY3029	*narK/nasA* family	31, Pa	2e-56	93
MARHY3030	*narX*	36, Ps	7e-107	99
MARHY3031	*narL*	57, Pa	2e-84	98
MARHY3053	*norC*	64, Ps	7e-66	100
MARHY3054	*norB*	77, Pa	0	96
MARHY3055	*ccoG*	26, Pd	1e-06	65
MARHY3056	*norD*	49, Ps	0	100
MARHY3057	*norE*	47, Ps	1e-45	98
MARHY3058	*norF* (see manuscript)*	60, Ps	0.006	32
MARHY3059	Hypothetical protein, no similarly	-	-	-
MARHY3060	*norQ*	72, Ps	2e-133	93
MARHY3061	Hypothetical protein, no similarly	-	-	-
MARHY3062	*nirF*	62, Pa	2e-171	95
MARHY3063	*nirC*	46, Ps	4e-21	72
MARHY3064	*nirS*	70, Pa	0	98
MARHY3065	*nirD*	53, Ps	3e-51	96
MARHY3066	*nirL*	53, Ps	3e-56	84
MARHY3067	*nirG*	59, Ps	8e-59	94
MARHY3068	*nirH*	60, Pa	9e-65	95
MARHY3069	*nirJ*	59, Ps	9e-168	94
MARHY3070	*nirE*	56, Ps	2e-93	91
MARHY3071	*nirN*	58, Ps	0	96
MARHY3072	Crp/Fnr family	32, Ps	2e-14	59
MARHY3073	*wrbA*	80, Ps	7e-117	100
MARHY3074	*yqfA*	44, Pa	5e-46	96
MARHY3075	*nnrS*	49, Ps	3e-50	64
MARHY3076	*narK2*	71, Ps	0	99
MARHY3077	Hypothetical protein (WP_049792410.1)	37, Pd	5e-19	83
MARHY3078	*narK/nasA* family	80, Ps	0	99
MARHY3079	Hypotherical protein (WP_003281670.1)	57, Ps	1e-53	97
MARHY3080	*narU (narK/nasA* family*)*	62, Pd	3e-166	94
MARHY0862	*fnrA*	53, Pa	2e-86	93

Note: Sequence homology with *Geobacillus stearothermophilus* (Gs) (taxid: 1422).

S3 - Score of the alignments of FNR and IHF motifs

Table S3 – FNR and IHF putative nucleotide motifs in the promoter regions of denitrification gene clusters of *M. hydrocarbonoclasticus*. PRODORIC database was used to identify the sequences.

PWM (species)	Promoter region	Sequence	Score
Fnr | *Escherichia coli* (strain K12) / Maximum score: 8.93	*narK*	**TTGA**GGATAG**TCAA**	7.14
	narGHJV	**TTGAT**CCCACA**CAA**	7.04
	narXL	**TTGA**CTATCC**TCAA**	7.92
	nirCF	**TT**AA**T**CAAT**ATCAA**	7.08
	nirSDLGHJEN	**TTGAT**ATTG**A**T**TAA**	7.30
	norCB3055DEF	**TTGAT**AATC**ATCAA**	8.47
	nosR	**TTGA**TTTTCC**TCAA**	8.16
	dnr	**TTGA**GCTAAG**TCAA**	7.27
IHF | *Escherichia* coli (strain K12) Maximum score: 7.67	*narK*	CCC**CAA**TGGT**TTA**TAA	6.39

Note: The nucleotides highlighted in bold match their consensus sequence. PWM- position weight matrix.

S4 – Composition of the Starkey oligoelement solution

The medium was supplemented with a Starkey oligoelement solution so that in the medium there would be 263 µM MgO, 20 µM $CaCO_3$, 22.3 µM $FeSO_4{\cdot}7H_2O$, 5 µM $ZnSO_4{\cdot}7H_2O$, 3.6 µM $MnSO_4{\cdot}H_2O$, 1 µM $CuSO_4{\cdot}5H_2O$, 3.2 µM CoSO4·7H2O, 0.97 µM BO_3H_3, 0.08 µM $Mo_7(NH_4)_6{\cdot}4H_2O$, 0.14 µM $Ni(NO_3)_2{\cdot}6H_2O$, 0.12 µM Na_2SeO_3.

S5 - Oxygen profile and schematic representation of the bioreactor

Oxygen profile measured in the liquid phase in the microaerobic growth in the bioreactor

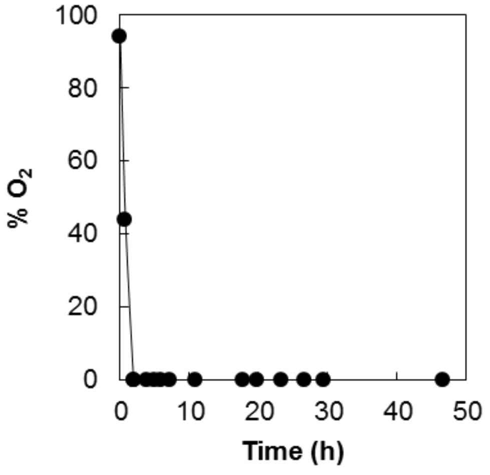

Figure S1 - Oxygen profile measured in the liquid phase of *M. hydrocarbonoclasticus* growth at pH 7.5 in the bioreactor during 48 h.

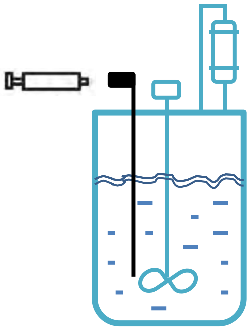

Figure S2 - Schematic representation of the bioreactor (blue). The location of sample collection is represented in black. Samples were collected from the bottom of the bioreactor through a syringe connected to a 0.22 µm filter to guarantee the sterility. Samples for RNA extraction were collected at flame and immediately frozen.

S6 - NO and N_2O reduction by the whole-cells - Data Analysis

The rate of NO and N_2O reduction by the whole-cells was indirectly measured through oxidation of methyl viologen. In the curve obtained, linear regressions were used to fit regions immediately before and after substrate addition. The slope of the fitting before substrate addition was subtracted to the slope obtained after substrate addition.

The rates were determined taking into consideration the calculated slope and the extinction coefficient of methyl viologen at 600 nm (ε_{600nm} = 11.4 mM^{-1} cm^{-1}) (Kristjansson & Hollocher 1980). We also consider that methyl viologen re-oxidation requires one electron while N_2O reduction involves two electrons and the reduction of two molecules of NO involves two electrons.

Note that in this assay, the subtraction of the slope prior to the addition of substrate is particularly important as other enzymes might also use the reduced methyl viologen, causing partial oxidation even before the addition of substrate.

Rates of NO and N_2O reduction were reported as micromoles of NO or N_2O reduced per minute per optical density ($\mu mol_{NO\ or\ N2O}$ min^{-1} OD^{-1}).

S7 – Genome organization of *M. hydrocarbonoclasticus* denitrification genes

A search in the genome of *M. hydrocarbonoclasticus* SP17 for genes involved in denitrification showed that several genes encoding proteins involved in the different steps of this pathway (see Figure S2). The *nosRZDFYL* gene cluster, containing the catalytic unit of N_2OR, encoded by *nosZ* (Philippot 2002; Zumft & Kroneck 2007). This gene cluster presents *nosR*, which encodes a transmembrane protein containing Fe-S centers and a periplasmic domain that binds a flavin (Cuypers et al. 1992; Wunsch & Zumft 2005). Although its function remains unclear, NosR seems to be important for *nosZ* transcription, as well as for enzyme full activity *in vivo*, as evidenced by mutational studies in *P. stutzeri* (Cuypers et al. 1992; Wunsch & Zumft 2005). The *nosD* gene encodes a protein that together with NosFY is proposed to form an ABC–transporter (NosDFY) and be involved in sulfur transport for CuZ center assembly (Wunsch et al. 2003; Zumft 2005a), while *nosL* encodes a putative copper chaperone (McGuirl et al. 2001).

Upstream *nos* cluster is the NaR gene cluster, *narLXKGHJV* (see Figure S2) containing the genes *narG*,

narH and *narV* (also designated as *narI*) that encode the α, β and γ subunits of the enzyme, respectively (Philippot 2002). The *narJ* gene encodes a chaperone-like component involved in the maturation and assembly of the enzyme complex (Lanciano et al. 2007), and *narXL* encode NarXL a two-component system. Several nark type genes have been identified in this genome, with one being associated with the *nar* cluster (*MARHY3029*). Two other *narK* genes are located upstream the *nir* operon, which correspond to *MARHY3076*, annotated as *narK2*, and *MARHY3078*, annotated as *narK*. Additionally, a *narU* (*MARHY3080*), encoding in *E. coli* a nitrate/nitrite transporter (Jia et al. 2009; Yan et al. 2013), is also found in this genome (see Figure S2).
Further upstream is *norBC*, encoding the two subunits of the short-chain membrane-bound *c*-NOR (see Figure S2). NorB (*MARHY3054*) is the catalytic subunit, while NorC (*MARHY3053*) is a small membrane bound *c*-type cytochrome functioning as an electron transfer subunit (Philippot 2002; Zumft 2005b). The accessory genes *norQ* (*MARHY3060*, annotated in other organisms as *napH*) and *norD* (*MARHY3056*), together with two other ORFs (*MARHY3057* and *MARHY3058*) are located upstream this operon. NorE and NorF, are predicted to be membrane associated, though their exact function remains unknown (Bergaust et al. 2014). It has been shown that NorEF are involved in denitrification as its inactivation slows nitrate reduction during denitrification, with accumulation of micromolar concentrations of nitric oxide (Bergaust et al. 2014; de Boer et al. 1996). In contrast, *norQ* and *norD* are always found linked to or in the vicinity of *norBC* (Zumft 2005b). The encoded proteins are suggested to be involved in the maturation of NorBC in either heme insertion, multisubunit assembly and/or insertion into the membrane (Zumft 1997; Zumft 2005b).
After the *norBC* genes, there is *nirFCSDLGHJEN*. The gene *nirS* encodes the cytochrome cd_1NiR, a homodimeric enzyme with *c*- and d_1-type hemes (Lopes et al. 2001). In recent years, it was identified that *nirDLGH* (Bali et al. 2011), *nirJ* (Bali et al. 2011), *nirE* (Zajicek et al. 2009) and *nirN* (Adamczack et al. 2014) encode enzymes involved in the biosynthesis of heme-d_1, all located in the cytoplasm, with the exception of NirN. NirF has been proposed to be a chaperone involved in the uptake and transport of dihydro-heme d_1 precursor from the cytoplasm to NirN located in the periplasm. The exact role of NirC remains unknown, but has been shown to be essential for the synthesis of heme-d_1 in *P. denitrificans* (Bali et al. 2014; de Boer et al. 1994). It is predicted to be a periplasmic *c*-type cytochrome, playing a role in the biosynthesis of heme-d_1 as an electron acceptor from NirN (Adamczack et al. 2014; Zajicek et al. 2009).
Finally, a gene annotated as *nnrS*, was identified upstream the *nir* cluster, which might be a membrane-bound NO sensor with a role in nitrosative stress, yet poorly explored (Stern et al. 2013).

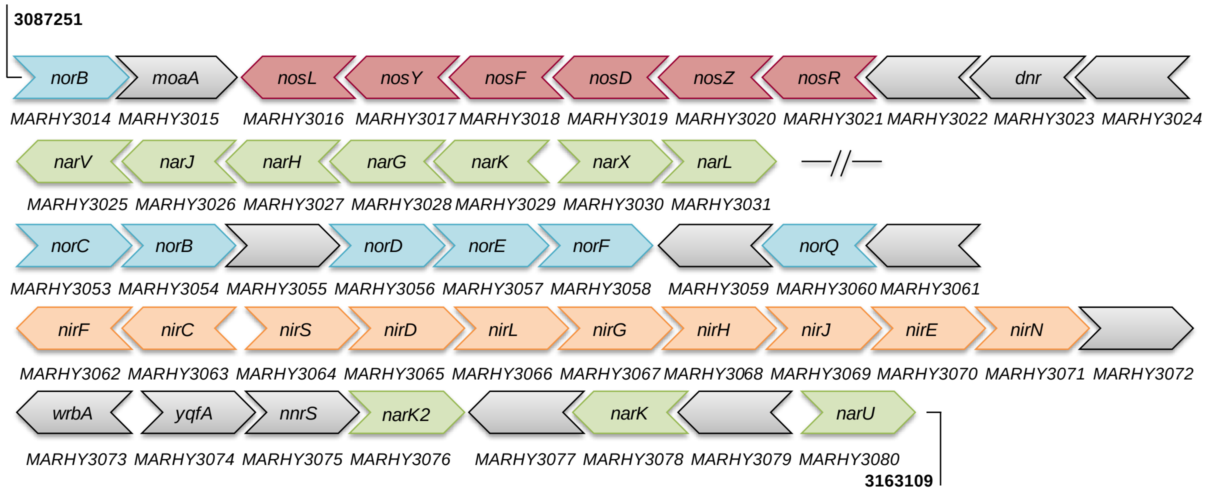

Figure S3 - Arrangement of denitrification genes in the genomic region 3087251-3163109 of *M. hydrocarbonoclasticus* SP17. Genes belonging to the nitrous oxide reductase cluster (*nos*) are shown in red, to the

nitrate reductase cluster (*nar*) are shown in green, to the nitrite reductase cluster (*nir*) are shown in orange and to the nitric oxide reductase cluster (*nor*) are shown in blue. Our own *in silico* analysis indicate that *MARHY3057* and *MARHY3058* are putative *norE* and *norF* homologues, respectively. Arrows show the direction of transcription. Genes are not drawn to scale.

References

Adamczack J, Hoffmann M, Papke U, Haufschildt K, Nicke T, Broring M, Sezer M, Weimar R, Kuhlmann U, Hildebrandt P, and Layer G. 2014. NirN protein from Pseudomonas aeruginosa is a novel electron-bifurcating dehydrogenase catalyzing the last step of heme d1 biosynthesis. *J Biol Chem* 289:30753-30762. 10.1074/jbc.M114.603886

Al-Attar S, and de Vries S. 2015. An electrogenic nitric oxide reductase. *FEBS Lett* 589:2050-2057. 10.1016/j.febslet.2015.06.033

Altschul SF, Madden TL, Schaffer AA, Zhang J, Zhang Z, Miller W, and Lipman DJ. 1997. Gapped BLAST and PSI-BLAST: a new generation of protein database search programs. *Nucleic Acids Res* 25:3389-3402.

Arai H, Igarashi Y, and Kodama T. 1995. The structural genes for nitric oxide reductase from Pseudomonas aeruginosa. *Biochim Biophys Acta* 1261:279-284.

Bali S, Lawrence AD, Lobo SA, Saraiva LM, Golding BT, Palmer DJ, Howard MJ, Ferguson SJ, and Warren MJ. 2011. Molecular hijacking of siroheme for the synthesis of heme and d1 heme. *Proc Natl Acad Sci U S A* 108:18260-18265. 10.1073/pnas.1108228108

Bali S, Palmer DJ, Schroeder S, Ferguson SJ, and Warren MJ. 2014. Recent advances in the biosynthesis of modified tetrapyrroles: the discovery of an alternative pathway for the formation of heme and heme d 1. *Cell Mol Life Sci* 71:2837-2863. 10.1007/s00018-014-1563-x

Bergaust LL, Hartsock A, Liu B, Bakken LR, and Shapleigh JP. 2014. Role of norEF in denitrification, elucidated by physiological experiments with Rhodobacter sphaeroides. *J Bacteriol* 196:2190-2200. 10.1128/JB.00003-14

Cantera JJ, and Stein LY. 2007. Molecular diversity of nitrite reductase genes (nirK) in nitrifying bacteria. *Environ Microbiol* 9:765-776. 10.1111/j.1462-2920.2006.01198.x

Cramm R, Pohlmann A, and Friedrich B. 1999. Purification and characterization of the single-component nitric oxide reductase from Ralstonia eutropha H16. *FEBS Lett* 460:6-10.

Cuypers H, Viebrock-Sambale A, and Zumft WG. 1992. NosR, a membrane-bound regulatory component necessary for expression of nitrous oxide reductase in denitrifying Pseudomonas stutzeri. *J Bacteriol* 174:5332-5339.

de Boer AP, Reijnders WN, Kuenen JG, Stouthamer AH, and van Spanning RJ. 1994. Isolation, sequencing and mutational analysis of a gene cluster involved in nitrite reduction in Paracoccus denitrificans. *Antonie Van Leeuwenhoek* 66:111-127.

de Boer AP, van der Oost J, Reijnders WN, Westerhoff HV, Stouthamer AH, and van Spanning RJ. 1996. Mutational analysis of the nor gene cluster which encodes nitric-oxide reductase from Paracoccus denitrificans. *Eur J Biochem* 242:592-600.

Ellis MJ, Grossmann JG, Eady RR, and Hasnain SS. 2007. Genomic analysis reveals widespread occurrence of new classes of copper nitrite reductases. *J Biol Inorg Chem* 12:1119-1127. 10.1007/s00775-007-0282-2

Gates AJ, Hughes RO, Sharp SR, Millington PD, Nilavongse A, Cole JA, Leach ER, Jepson B, Richardson DJ, and Butler CS. 2003. Properties of the periplasmic nitrate reductases from Paracoccus pantotrophus and Escherichia coli after growth in tungsten-supplemented media. *FEMS Microbiol Lett* 220:261-269.

Ghiglione JF, Philippot L, Normand P, Lensi R, and Potier P. 1999. Disruption of narG, the gene encoding the catalytic subunit of respiratory nitrate reductase, also affects nitrite respiration in Pseudomonas fluorescens YT101. *J Bacteriol* 181:5099-5102.

Hendriks J, Oubrie A, Castresana J, Urbani A, Gemeinhardt S, and Saraste M. 2000. Nitric oxide reductases in bacteria. *Biochim Biophys Acta* 1459:266-273.

Jia W, Tovell N, Clegg S, Trimmer M, and Cole J. 2009. A single channel for nitrate uptake, nitrite export and nitrite uptake by Escherichia coli NarU and a role for NirC in nitrite export and uptake. *Biochem J* 417:297-304. 10.1042/bj20080746

Jungst A, Braun C, and Zumft WG. 1991. Close linkage in Pseudomonas stutzeri of the structural genes for respiratory nitrite reductase and nitrous oxide reductase, and other essential genes for denitrification. *Mol Gen Genet* 225:241-248.

Kristjansson JK, and Hollocher TC. 1980. First practical assay for soluble nitrous oxide reductase of denitrifying bacteria and a partial kinetic characterization. *J Biol Chem* 255:704-707.

Lanciano P, Vergnes A, Grimaldi S, Guigliarelli B, and Magalon A. 2007. Biogenesis of a respiratory complex is orchestrated by a single accessory protein. *J Biol Chem* 282:17468-17474. 10.1074/jbc.M700994200

Lopes H, Besson S, Moura I, and Moura JJ. 2001. Kinetics of inter- and intramolecular electron transfer of Pseudomonas nautica cytochrome cd1 nitrite reductase: regulation of the NO-bound end product. *J Biol Inorg Chem* 6:55-62.

McGuirl MA, Bollinger JA, Cosper N, Scott RA, and Dooley DM. 2001. Expression, purification, and characterization of NosL, a novel Cu(I) protein of the nitrous oxide reductase (nos) gene cluster. *J Biol Inorg Chem* 6:189-195.

McWilliam H, Li W, Uludag M, Squizzato S, Park YM, Buso N, Cowley AP, and Lopez R. 2013. Analysis Tool Web Services from the EMBL-EBI. *Nucleic Acids Res* 41:W597-600. 10.1093/nar/gkt376

Nojiri M, Xie Y, Inoue T, Yamamoto T, Matsumura H, Kataoka K, Deligeer, Yamaguchi K, Kai Y, and Suzuki S. 2007. Structure and function of a hexameric copper-containing nitrite reductase. *Proc Natl Acad Sci U S A* 104:4315-4320. 10.1073/pnas.0609195104

Philippot L. 2002. Denitrifying genes in bacterial and Archaeal genomes. *Biochim Biophys Acta* 1577:355-376.

Simon J, Einsle O, Kroneck PM, and Zumft WG. 2004. The unprecedented nos gene cluster of Wolinella succinogenes encodes a novel respiratory electron transfer pathway to cytochrome c nitrous oxide reductase. *FEBS Lett* 569:7-12. 10.1016/j.febslet.2004.05.060

Stern AM, Liu B, Bakken LR, Shapleigh JP, and Zhu J. 2013. A novel protein protects bacterial iron-dependent metabolism from nitric oxide. *J Bacteriol* 195:4702-4708. 10.1128/JB.00836-13

Viebrock A, and Zumft WG. 1988. Molecular cloning, heterologous expression, and primary structure of the structural gene for the copper enzyme nitrous oxide reductase from denitrifying Pseudomonas stutzeri. *J Bacteriol* 170:4658-4668.

Wunsch P, Herb M, Wieland H, Schiek UM, and Zumft WG. 2003. Requirements for Cu(A) and Cu-S center assembly of nitrous oxide reductase deduced from complete periplasmic enzyme maturation in the nondenitrifier Pseudomonas putida. *J Bacteriol* 185:887-896.

Wunsch P, and Zumft WG. 2005. Functional domains of NosR, a novel transmembrane iron-sulfur flavoprotein necessary for nitrous oxide respiration. *J Bacteriol* 187:1992-2001. 10.1128/jb.187.6.1992-2001.2005

Yan H, Huang W, Yan C, Gong X, Jiang S, Zhao Y, Wang J, and Shi Y. 2013. Structure and mechanism of a nitrate transporter. *Cell Rep* 3:716-723. 10.1016/j.celrep.2013.03.007

Zajicek RS, Bali S, Arnold S, Brindley AA, Warren MJ, and Ferguson SJ. 2009. d(1) haem biogenesis - assessing the roles of three nir gene products. *FEBS J* 276:6399-6411. 10.1111/j.1742-4658.2009.07354.x

Zumft WG. 1997. Cell biology and molecular basis of denitrification. *Microbiol Molec Biol Rev* 61:533-616.

Zumft WG. 2005a. Biogenesis of the bacterial respiratory CuA, Cu-S enzyme nitrous oxide reductase. *J Mol Microbiol Biotechnol* 10:154-166. 10.1159/000091562

Zumft WG. 2005b. Nitric oxide reductases of prokaryotes with emphasis on the respiratory, heme-copper oxidase type. *J Inorg Biochem* 99:194-215. 10.1016/j.jinorgbio.2004.09.024

Zumft WG, and Kroneck PM. 2007. Respiratory transformation of nitrous oxide (N2O) to dinitrogen by Bacteria and Archaea. *Adv Microb Physiol* 52:107-227. 10.1016/s0065-2911(06)52003-x
